# Identification and Validation of Three PDAC Subtypes and Individualized GSVA Immune Pathway-Related Prognostic Risk Score Formula in Pancreatic Ductal Adenocarcinoma Patients

**DOI:** 10.1155/2021/4986227

**Published:** 2021-12-27

**Authors:** Deyu Zhang, Meiqi Wang, Lisi Peng, Xiaoli Yang, Keliang Li, Hua Yin, Chuanchao Xia, Fang Cui, Haojie Huang, Zhendong Jin

**Affiliations:** ^1^Department of Gastroenterology, Changhai Hospital, Shanghai, China; ^2^Department of Gastroenterology, First Affiliated Hospital of Zhengzhou University, Zhengzhou, China

## Abstract

**Background:**

With the progress of precision medicine treatment in pancreatic ductal adenocarcinoma (PDAC), individualized cancer-related medical examination and prediction are of great importance in this high malignant tumor and tumor-immune microenvironment with changed pathways highly enrolled in the carcinogenesis of PDAC.

**Methods:**

High-throughput data of pancreatic ductal adenocarcinoma were downloaded from Gene Expression Omnibus (GEO) and The Cancer Genome Atlas (TCGA) database. After batch normalization, the enrichment pathway and relevant scores were identified by the enrichment of immune-related pathway signature using gene set variation analysis (GSVA). Then, cancerous subtype in TCGA and GEO samples was defined through the NMF methods by cancertypes packages in R software, respectively. Subsequently, the significance between the characteristics of each TCGA sample and cancer type and the significant prognosis-related pathway with risk score formula is calculated through t-test and univariate Cox analysis. Next, the prognostic value of gained risk score formula and each significant prognosis-related pathway were validated in TCGA and GEO samples by survival analysis. The pivotal hub genes in the enriched significant prognosis-related pathway are identified and validated, and the TIMER database was used to identify the potential role of hub genes in the PDAC immune environment. The potential role of hub genes is promoting the transdifferentiation of cancer-associated fibroblasts.

**Results:**

The enrichment pathway and relevant scores were identified by GSVA, and 3 subtypes of pancreatic ductal adenocarcinoma were defined in TCGA and GEO samples. The clinical stage, tumor node metastasis classification, and tumor grade are strongly relative to the subtype above in TCGA samples. A risk formula about GSVA significant pathway “GSE45365_WT_VS_IFNAR_KO_CD11B_DC_MCMV_INFECTION_DN ∗ 0.80 + HALLMARK_GLYCOLYSIS ∗ 16.8 + GSE19888_CTRL_VS_T_CELL_MEMBRANES_ACT_MAST_CELL_DN ∗ 14.4” was identified and validated in TCGA and GEO samples through survival analysis with significance. DCN, VCAN, B4GALT7, SDC1, SDC2, B3GALT6, B3GAT3, SDC3, GPC1, and XYLT2 were identified as hub genes in these GSVA significant pathways and validated in silico.

**Conclusions:**

Three pancreatic ductal adenocarcinoma subtypes are identified, and an individualized GSVA immune pathway score-related prognostic risk score formula with 10 hub genes is identified and validated. The predicted function of the 10 upregulated hub genes in tumor-immune microenvironment was promoting the infiltration of cancer-associated fibroblasts. These findings will contribute to the precision medicine of pancreatic ductal adenocarcinoma treatment and tumor immune-related basic research.

## 1. Introduction

Pancreatic ductal adenocarcinoma (PDAC) is a life-threatening disease with the lowest survival rates among major cancers, and its mortality rate per year is increasing from 9th to 7th [1]. Positive results of computed tomography (CT) often only occur on terminal patients with PDAC, with a delayed diagnosis and poor prognosis of patients [2]. Additionally, the poor prognosis of patients with PDAC is also due to the high recurrence rate and early distant metastasis [3]. Aiming to prompt diagnosis and treatment, some advanced effective procedures have been put forward, including nucleic acid in circulating cancer cells, long noncoding RNA in an extracellular vesicle, and some pivotal clinical characteristics [4–6]. Besides these, some other individualized diagnostic methods based on sequencing and key pathway need to be identified.

Several original gene-related diagnostic and prognostic signatures have been developed to estimate a clinical outcome and instruct precise treatment of various types of patients with cancer, including pulmonary carcinoma [7] and gastric cancer [8]. Meanwhile, some miRNA-based diagnostic and prognostic signatures also show great utilization potentialities in prognosis prediction and treatment, such as the preferable predictive value of screened miRNA in lymph-gland tumor [9] and a lncRNA-based formula developed for renal cancer [10]. As described above, most of the current research studies focus on the predicting potential of a cluster of screened genes, namely, lncRNAs and miRNAs. The research studies on carcinogenesis-related pathways need to be clarified, and screening a cluster of prognostic-related pathways can facilitate in cancer treatment.

The gene set variation analysis (GSVA) is a nonparametric, unsupervised algorithm. The GSVA does not require pregrouping of samples and can calculate enrichment scores for specific sets of genes in each sample. Additionally, GSVA transforms gene expression data from an expression matrix of individual genes as traits to an expression matrix of specific gene sets as traits. The results of gene enrichment were quantified by GSVA, which can be more convenient for follow-up statistical analysis [11]. The GSVA method has been well performed on some research studies that focus on pathology mechanism, such as GSVA was used on calculating T-cell receptors and ligands related to T-cell failure and indicating T-cell apoptosis and activated expression of cell cycle genes in the process of neuroblastoma [12].

In this study, we used the NMF method to identify PDAC subtypes and developed and verified a GSVA-based immune pathway formula aiming to predict the prognostic clinical outcomes of patients and identify the pivotal pathway. Then, the hub genes in these pivotal pathways are identified and the potential roles in the tumor-immune microenvironment were predicted. Our findings could facilitate the precise treatment and basic research of PDAC.

## 2. Materials and Methods

### 2.1. Data Source

The gene expression matrixes with paired clinical data are downloaded in The Cancer Genome Atlas (TCGA) database (https://cancergemo.me.nih.gov/) named TCGA-PDAC (TCGA PDAC: 4 paracarcinoma tissue and 178 cancers with survival data) and two datasets in Gene Expression Omnibus (GEO) database (http://www.ncbi.nlm.nih.gov/geo/), GSE28735, and GSE62452. (GSE28735 : 45 paracarcinoma tissue and 45 carcinoma tissue with survival data. GSE62452 : 61 paracarcinoma tissue and 69 carcinoma tissue with survival data.)

### 2.2. Batch Normalization

The batch effect is part of the measurement results because of the different experimental conditions. The purpose of correcting the batch effect is to reduce the irrelevant differences between batches and to identify the differences between different biological states. To remove the impact of batch effect between GSE28735 and GSE62452, the SVA package was used in R software [13].

### 2.3. Gene Set Variation Analysis

Gene set variation analysis (GSVA) is a nonparametric unsupervised analysis method mainly used to evaluate the gene set enrichment results of sequencing. The expression matrix of genes is transformed into the expression matrix of pathways in different samples to evaluate different metabolic pathways enriched in different samples. It mainly is to explain the causes of phenotypic differences from a bioinformatic perspective. In this study, we perform GSVA using GSVA package in R software, and an immune-related gene set c7.immunesigdb_HALLMARK was used in GSVA.

### 2.4. Cancer Subtype Identification

CancerSubtypes package [14] in R software was exerted on TCGA pathway expression matrix with survival data and validated on combined GEO data (GSE28735 and GSE62452). Non-negative matrix factorization (NMF) is a powerful method for reducing the dimension of data and has a wide range of applications in the identification of functional part for complex data with several dimensions. We used the “NMF” and “factoextra” packages to perform NMF on TCGA and GEO datasets above to further confirm the differentiation among the acquired PDAC subtypes.

### 2.5. Survival Analysis

Survival analysis is the method to analyze and infer the survival time of patients with PDAC in different groups based on clinical data from TCGA and GEO, aiming to study the relationship among survival time, patients' outcomes, and various influencing factors. In our study, survival analysis was performed using the survival package in R software.

### 2.6. Visualization and Data Statistics

All visualization and data statistics were exerted in R software. In particular, complexHeatmap package was used on TCGA-PDAC data to identify the classification of subtypes. Differential analysis of gene sets was calculated using the limma package. Then, LASSO regression was exerted using the Glmnet package. The ClusterProfiler package in R software was used to identify key KEGG pathway in the pivotal immune-related pathway, and Cytohubba package in Cytoscape software was used to screen hub genes in pivotal immune-related pathway above.

### 2.7. Database Manipulation

TIMER database (Tumor Immune Estimation Resource:https://cistrome.shinyapps.io/timer/) includes “EPIC,” “Mcpcounter,” “Xcell,” and “Tide.” TIMER was used to estimate the tumor immune infiltration by combining the algorithm above and TCGA sequencing data. The GEPIA (Gene Expression Profiling Interactive Analysis) is another database containing TCGA tumor sequencing data and GTEx normal tissue sequencing data, and it was used to identify the expression of hub genes among pancreatic tumor tissue and common tissue.

## 3. Results

### 3.1. Transitional Signatures of Immune-Related Pathway among PDAC and Paracarcinoma Tissue

**Figure 1:**
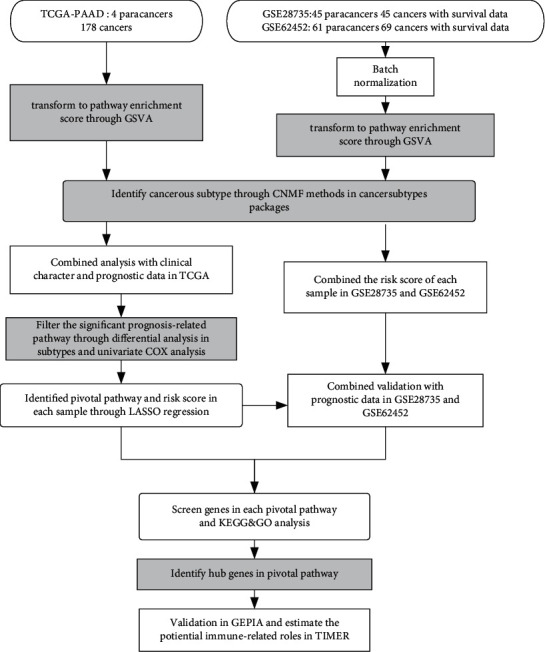
Flow chart of our study.

Figure 1 illustrates the procedure of our study. First, to identified the transitional signatures of immune-related pathway among PDAC and paracarcinoma tissues, PDAC sequencing data were screened and downloaded in TCGA (TCGA PDAC: 4 paracarcinoma tissues and 178 cancerous tissues with survival data) and GEO databases (GSE28735 : 45 paracancers and 45 cancers with survival data. GSE62452 : 61 paracancers and 69 cancers with survival data). Then, the hallmarks get set, and immune-related pathways (“c7.immunesigdb_HALLMARK,” contained 4922 gene sets) were downloaded from GSVA (https://www.gseamsigdb.org/gsea/msigdb/index.jsp).

**Figure 2:**
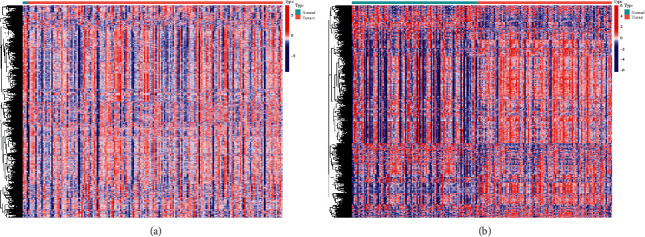
Heatmap of TCGA and GEO data. (a) Enrichment score of immunologic and hallmark genes between cancers and paracarcinoma tissues in TCGA-PDAC. (b) Enrichment score of immunologic and hallmark gene cancers and paracarcinoma tissues in GSE28735 and GSE62452.

GSVA was used on the TCGA-PDAC cohort, and the enrichment score is clustered and visualized (Figure 2(a)). After batch normalization of GSE28735 and GSE62452, GSVA was exerted in this GEO cohort (Figure 2(b)). The screened gene sets appear to classify cancers and paired paracarcinoma tissues into several subtypes.

### 3.2. Identification of PDAC Subtypes and the Relevance between PDAC Subtype and Clinical Characteristics

**Figure 3:**
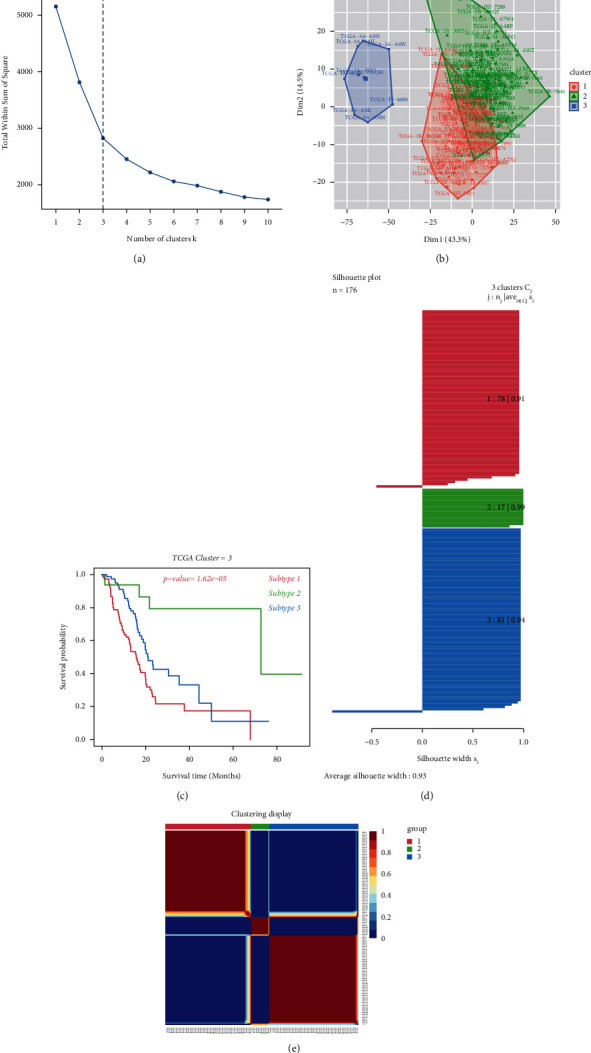
(a) Identification of best cutoff of cluster. (b) The distribution of each cluster by PCA method in factoextra package. (c) Survival analysis of TCGA-PDAC by clusters. (d) Identification of the value of grouping by silhouette width plots. (e) Visualization of each cluster using the NMF method.

After the expression of immune-related pathway and gene sets in two PDAC cohorts had been manipulated (4,922 gene sets), cancer subtypes are identified by the “CancerSubtypes” package in R software. By using the NMF method, *K* = 3 had been identified as the best cutoff number of cluster, which means PDAC could be separated into 3 subtypes by the GSVA immune-related pathway score of each sample (Figure 3(a)). Then, Figure 3(b) shows the three divided clusters. Combining with clinical data, the different prognosis among the three PDAC subtypes was identified (Figure 3(c)). In particular, cluster 1 shows a significant favorable prognosis. Cluster 2 displays a medium prognosis among the 3 clusters, and cluster 3 reveals the poorest prognosis. Cluster display plot and silhouette width plot show high credibility subtype identification (0.93 average silhouette width, Figures 3(e) and 3(d)). Subsequently, these 3 subtype separation methods were validated on GEO cohorts (Supplementary Materials (available here)).

**Figure 4:**
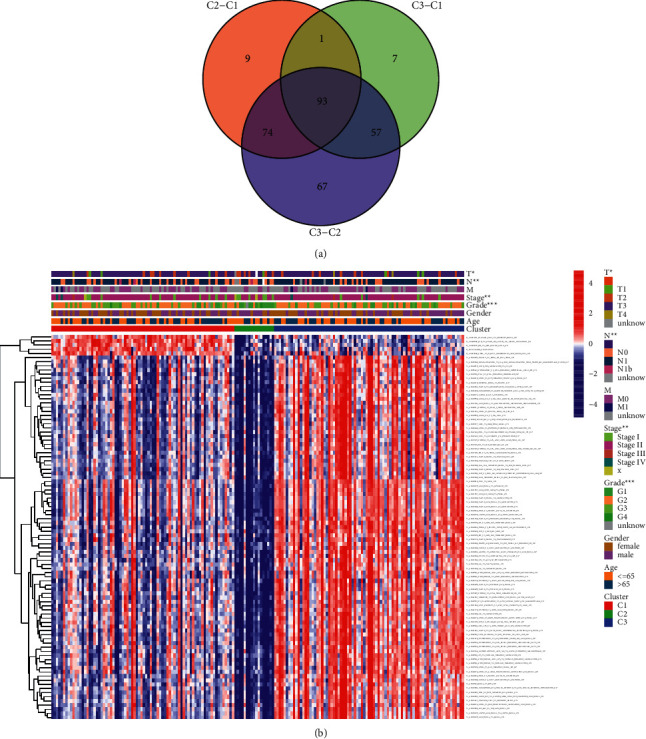
(a) The common gene sets and pathways among the 3 subtypes. (b) The clinical characteristic and the expression of 93 intersected gene sets and pathways among the 3 different PDAC subtypes.

Then, the relevance between clinical characteristic and each divided subtype is confirmed in the TCGA-PDAC cohort. In brief, patients in subtype 1 are often accompanied by high clinical stage, pathologic grade (G), and bad TN classification. Patients in subtype 2 are often together with moderate clinical stage, pathologic grade (G), and TN classification, and patients in subtype 3 often have good to moderate clinical stage, pathologic grade (G), and TN classification with statistical significance. The differently expressed pathways with significance by pairwise comparison were enriched and calculated in each cluster (adjusted *P* value <0.05). A total of 93 significant pathways were enriched as common differently expressed pathways in the 3 subtypes (Figure 4(b)). The details of the clinical relevance among subtypes are shown in Table 1. The patient status, R0 resection rate, tumor size, TMN classification, the WHO classification, pathological grade, and alcohol history among these 3 subtypes show great differentiation (*P* < 0.05). Patients in subtype 1 are inclined to have the worst condition in these clinical characteristic, while patients in subtype 2 group tend to get the best condition.

### 3.3. Calculation and Validation of Prognostic Signaling Pathway and Gene Set-Based Formula

**Figure 5:**
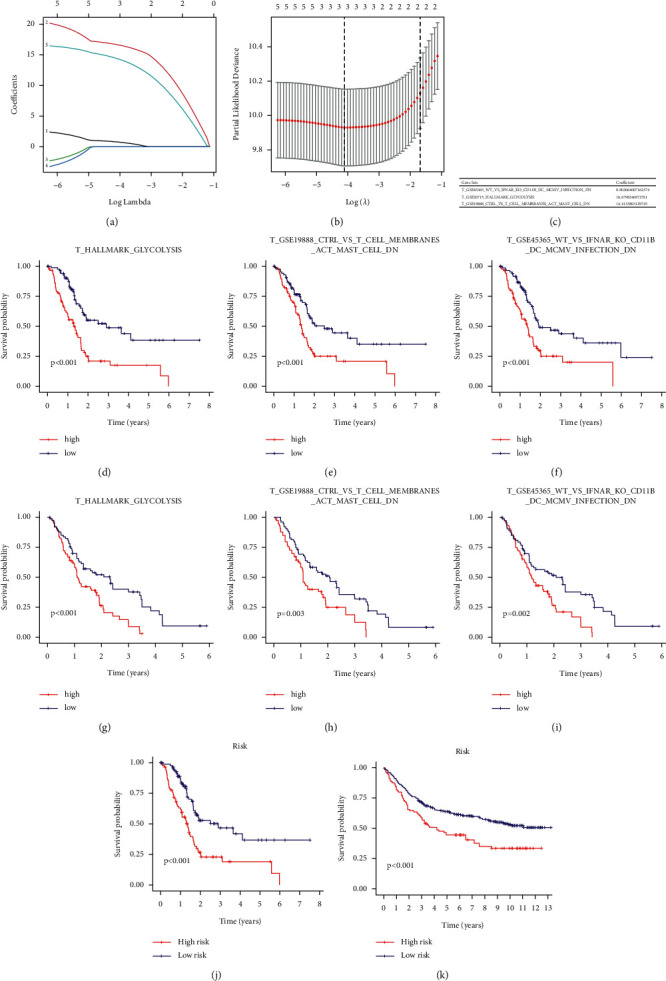
(a) Based on TCGA-PDAC data, absolute shrinkage and selection operator (LASSO) coefficient profiles were exerted. (b) Best penalization coefficient (*λ*) by threefold validation according to partial likelihood deviance. (c) The significant gene sets and pathways after LASSO regression with the risk coefficient. (d–f) Survival analysis of each gene set and pathway on TCGA-PDAC cohort. (g–i) Survival analysis of each gene set and pathway on the GSE28735 and GSE62452 cohorts. (j) Survival analysis of the calculated risk formula on TCGA-PDAC cohort. (k) Survival analysis of the calculated risk formula on GSE28735 and GSE62452 cohort.

Combining with the survival data of patients, univariable Cox regression analysis was used to screen on 93 intersected gene sets and pathways. Four gene sets and 1 pathway were screened, including GSE45365_WT_VS_IFNAR_KO_CD11B_DC_MCMV_INFECTION_DN, GSE20715_0H_VS_48H_OZONE_LUNG_DN, GSE13411_PLASMA_CELL_VS_MEMORY_BCELL_UP, GSE19888_CTRL_VS_T_CELL_MEMBRANES_ACT_MAST_CELL_DN and GLYCOLYSIS pathway (Table 2). To identify the pivotal part of these gene sets, LAASO regression is exerted in Figures 5(a) and 5(b), and 2 gene sets and 1 pathway were identified with risk coefficient (Figure 5(c)). Their prognostic value was then validated by survival analysis based on PDAC-TCGA (Figures 5(d)–5(f)) and GSE28735 and GSE62452 (Figures 5(g)–5(i)). The formula was constructed based on the screened gene sets and risk coefficient: Risk score = GSE45365_WT_VS_IFNAR_KO_CD11B_DC_MCMV_INFECTION_DN ∗ 0.80 + HALLMARK_GLYCOLYSIS ∗ 16.8 + GSE19888_CTRL_VS_T_CELL_MEMBRANES_ACT_MAST_CELL_DN ∗ 14.4. The risk score based on the formula was calculated. Then, survival analysis was executed on the TCGA-PDAC cohort and validated GSE28735 and GSE62452 cohorts (Figures 5(j) and 5(k)).

### 3.4. Identification of Significant Pathway and Hub Genes in the Screened Prognosis-Related Gene Sets

**Figure 6:**
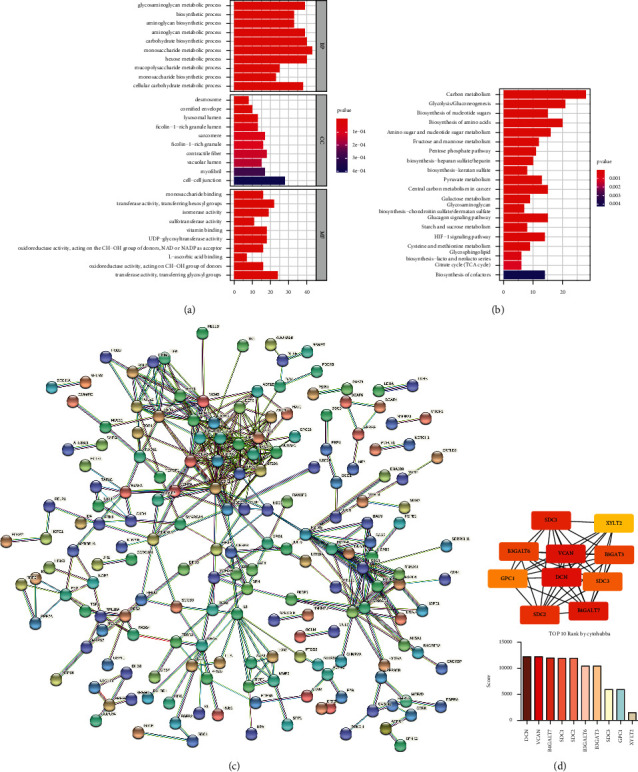
(a) Gene ontology (GO) analysis shows enriched GO term. (b) Kyoto Encyclopedia of Genes and Genomes (KEGG) analysis shows enriched signaling pathways. (c) Protein-protein interaction analysis. (d) Hub genes were found and ranked by Cytoscape and Cytohubba.

Genes in the 3 prognosis-related gene sets and pathways were extracted, and gene ontology enrichment analysis was performed. Several cancer-related metabolism GO term and “tight junction” are identified in Figure 6(a). Then, KEGG enrichment analysis is exerted as Figure 6(b). Some pivotal pathways are involved, including the “HIF-1 signaling pathway” and “pyruvate metabolism” (Figure 6(b)). Additionally, we performed protein-protein interaction analysis, and the top 10 ranked hub genes are identified by enriched scores, including DCN, VCAN, B4GALT7, SDC1, SDC2, B3GALT6, B3GAT3, SDC3, GPC1, and XYLT2.

### 3.5. Validation of the Hub Genes and Estimating Their Potential Roles in Promoting the Infiltration of Cancer-Associated Fibroblasts (CAFs) in Tumor Environments

**Figure 7:**
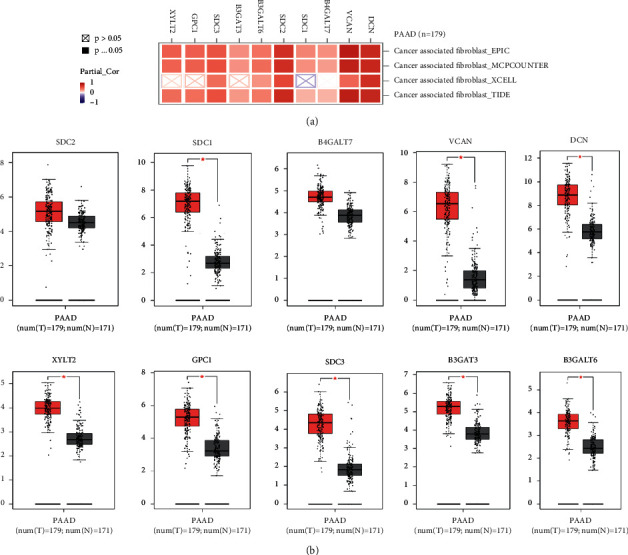
(a) The 10 hub genes are enriched in promoting the infiltration of cancer-associated fibroblasts (CAFs) using 4 algorithms in the TIMER database. (b) Validating the differential expression of the 10 hub genes in the GEPIA database.

Then, the potential roles of the screened 10 hub genes in tumor-immune microenvironment were predicted on the TIMER database. Among all the tumor-related immune cells, the 10 hub genes were significantly enriched in cancer-associated fibroblasts (CAFs) (Figure 7). Finally, upregulation of the 10 hub genes was validated in the GEPIA database. All 10 hub genes are upregulated in cancer tissue compared to normal pancreas. Eight hub genes, including DCN, VCAN, SDC1, B3GALT6, B3GAT3, SDC3, GPC1, and XYLT3, are significantly upregulated in PDAC tissue.

## 4. Discussion

As a fatal malignant tumor, PDAC causes a huge social health burden and patients could neither be directly diagnosed in the early stage nor be accurately predicted the clinical outcome. At this point, the prognostic marker is in great need for the treatment of patients with PDAC [15]. In this study, we used bioinformatic and statistical methods to screen the biomarkers with great prognostic sensitivity. Through the combination of the GSVA method, NMF algorithm, and the obtained sequencing data from the TCGA database, we put forward a new grouping method to predict the prognosis of patients with PDAC and validate in GEO samples. In fact, several studies have manipulated on analyzing the subtype of PDAC using the machine-learning algorithm. Wu et al. reported a procedure to cluster PDAC samples into 2 subtypes based on TCGA sequencing data. However, the K-means algorithm was used in the study of Sinkala Musalula, and in our study, we used the NMF algorithm to calculate the sequencing data, which could be more sensitive to identify the main characteristic [16], and silhouette width analysis shows high credibility subtype identification (0.93 average silhouette width). Zhang et al. [17] have established a GSVA-related miRNA and mRNA signature. However, the subtype was not analyzed in their study, and the significant gene sets and pathways in their study were not screened by the subtype of PDAC, which could compromise the conclusion. After the subtype identification, the clinical data, including patient status, tumor classification, and carcinogenic habit, were calculated and our clustered cancer subtypes showed robust competency on distinguishing tumor classification. After common gene sets and pathways were identified among the 3 PDAC subtypes and LASSO regression, the glycolysis pathway and other two immune pathways were identified. Some previous research studies have focused on the glycolysis pathway in the carcinogenesis of PDAC. Dai Shang-nan et al. reported that glycolysis promotes the progression of pancreatic cancer and reduces cancer cell sensitivity to gemcitabine [18]. Zhang et al. established a glycolysis-related prognostic formula to predict the chemosensitivity of patients with PDAC [19]. The result from our study also illustrates the emergency role of the glycolysis pathway in PDAC using GSVA.

By analyzing the hub genes in the screened gene sets and pathways, the HIF-1 signaling pathway was identified as a distinct pathway, and the immune environment relevance shows that cancer-associated fibroblasts are significantly enriched in the upregulated hub genes. PDAC is a tumor with a high level of fibrosis, and a dense fibrotic matrix takes up 90% of tumorous bulk [20]. The origin of the fibrotic matrix has been widely studied, and cancer-associated fibroblasts (CAFs) have been illustrated to play an important role in tumorous fibrosis [21]. Chen et al. separated CAFs and normal fibroblasts (NFs) obtained from PDAC tissue, and CAFs showed strong glucose absorption and lactic acid production [22]. Compared with NFs, pyruvate kinase M2 (PKM2) in CAFs is upregulated. The finding hints that metabolic reprogramming occurs in CAFs with significant high expression of glucose. Some studies hint that the HIF-1 signaling pathway participates in tumorous fibrosis. Goodwin et al. demonstrated that some pivotal kinase in glucose could be activated by the HIF-1 pathway [23]. Our finding illustrates that glycolysis is a pivotal biologic process in PDAC with significant prognostic value, and the potential upstream mechanism is associated with the activation of the HIF-1 pathway. The downstream mechanism could be abnormally activated glucose promote the transdifferentiation of CAFs.

Another light spot of our study is some found hub genes, namely, DCN, VCAN, B4GALT7, SDC1, SDC2, B3GALT6, B3GAT3, SDC3, GPC1, and XYLT2. Some cancer-related mechanism of these molecules has been reported: DCN encodes decorin, a class I SLRP that participates in collagen fibrillogenesis. In carcinogenesis, decorin could promote the development of cancer as a pan tyrosine kinase inhibitor [24]. Zhang et al. recently reported DCN is able to promote tumor invasion and migration in pancreatic cancer cells [25]. VCAN is a member of a large chondroitin sulfate proteoglycan family with hyaluronate-binding capacities. VCAN participates in cancer-related intercellular substance formation to promote tumor cell proliferation and invasion in multiple types of cancers [26,27]. VCAN was also reported to have an important role in the invasion phenotypes of PDAC [24]. B4GALT7, B3GALT6, and B3GAT3 encode three subtypes of galactosyltransferase. Galactosyltransferases are related to glycosaminoglycans and to proteoglycans in multiple tissues, including cancer development [28,29]. Syndecans are a family of transmembrane glycoproteins, and the syndecan-medicated calcium metabolism is associated with cell adhesion, the dysfunction of syndecans is a pivotal biologic process in tumor development [30]. In particular, SDC1 is also reported to facilitate tumor invasion via stimulating the EMT pathway in pancreatic cancer cells [31]. SDC2 can promote epithelial-mesenchymal transition in colon cancer [32]. SDC3 could also promote melanoma tumors through the regulation of the HIF pathway [33]. Interestingly, the significant differential expression has been screened and these genes are identified as the hub genes in the glycolysis pathway after GSVA in PDAC, which needs further research on their behaviors in carcinogenesis. The full name of XYL2 is xylosyltransferase 2, and XYL2 participated in the proteoglycan (PG) biochemistry of multiple tumor tissues [34]. Upregulation of GPC1 is associated with a unfavorable prognosis of PDAC [35]. Comprehensively, the findings are mostly focusing their potential roles in cancer signaling transduction. Based on this study, the mechanism of these upregulated hub genes on restraining tumor immunity and promoting transdifferentiation of CAFs and other verification based on clinical samples needs to be studied further.

## 5. Conclusions

In this study, we established a new clustering method of PDAC subtype by machine-learning method and verified its functional competency in two cohorts from the database. Then, we build a GSVA-based prognostic formula by subtype clustering, Cox, and LASSO regression and validated its predicting competency on the relevance of TCGA-PDAC clinical characteristics and survival data in two combined GEO datasets after batch normalization. Additionally, 10 upregulated hub genes were identified and validated in three significant GSVA-based gene sets, and the function of the gene sets and hub genes in PDAC tumor immunity was predicted as promoting the transdifferentiation of CAFs.

## Figures and Tables

**Table 1:** The relevance among subtypes and clinical data in TCGA-PDAC.

	Subtype 1 (*n* = 78)	Subtype 2 (*n* = 17)	Subtype 3 (*n* = 81)	*P* value
Gender				0.848
Male	40	11	45	
Female	38	6	36	
Age (average age)				0.763
>65	40 (73.5)	6	37 (74.5)	
≦65	38 (60.0)	11	44 (56.5)	
Status (dead, %)	34 (43.6)	3 (17.6)	20 (24.7)	<0.01
Residual tumor (R0, %)	41 (52.6)	13 (76.5)	52 (64.2)	<0.01
Tumor size (≤4 cm) (%)	45 (57.7)	11 (64.7)	58 (61.7)	0.236
*TNM classification (%)*				
T1	3 (3.8)	1 (5.9)	3 (3.7)	<0.01
T2	9 (11.5)	6 (35.3)	9 (11.1)	
T3&4	66 (84.6)	8 (47.1)	69 (85.1)	
Tx (missing)	0	2 (11.8)	0	
M0	35 (44.9)	5 (29.4)	40 (49.4)	0.893
M1	2 (2.6)	0	2 (2.5)	
Mx (missing)	41 (52.6)	0	39 (48.1)	
N0	24 (30.8)	7 (41.2)	18 (22.2)	0.02
N1	54 (69.2)	7 (41.2)	61 (75.3)	
Nx (missing)	0	3 (17.6)	2 (2.5)	
*WHO classification*				
Stage I	10 (12.8)	6 (35.3)	5 (6.2)	0.02
Stage II	64 (82.1)	9 (52.9)	72 (88.9)	
Stage III and IV	4 (5.1)	0	4 (4.9)	
Stage x (missing)	0	2 (11.8)	0	
*Pathological grade*				
G1	7 (9.0)	11 (64.7)	12 (14.8)	0.02
G2	44 (56.4)	2 (11.8)	48 (59.3)	
G3 and G4	27 (34.6)	3 (17.6)	21 (25.9)	
Gx (missing)	0	1 (5.9)	0	
Alcohol history (YES, %)	49 (62.8)	6 (35.3)	40 (49.4)	0.04
Smoking history (YES, %)	24 (30.8)	5 (29.4)	30 (37.0)	0.248

**Table 2:** The screened gene sets and pathways after univariable Cox regression analysis.

Gene sets and pathways	HR	HR.95L	HR.95H	*P* value
T_GSE45365_WT_VS_IFNAR_KO_CD11B_DC_MCMV_INFECTION_DN	5.03	4.94	5.23	0.003
T_GSE20715_0H_VS_48H_OZONE_LUNG_DN	2.92	3.04	1.17	0.005
T_GSE13411_PLASMA_CELL_VS_MEMORY_BCELL_UP	3.73	1.92	7.74	0.014
T_HALLMARK_GLYCOLYSIS	8.97	8.43	9.25	0.001
T_GSE19888_CTRL_VS_T_CELL_MEMBRANES_ACT_MAST_CELL_DN	2.89	2.15	3.87	0.002

## Data Availability

The PAAD dataset was downloaded from the TCGA database (https://tcga-data.nci.nih.gov/tcga/), and GSE28735 and GSE62452 were downloaded from the GEO database (http://www.ncbi.nlm.nih.gov/geo/).

## Supplementary Materials

Supplementary Materials Supplementary Figure 1: (A) identification of best cutoff of cluster. (B) The distribution of each cluster by the PCA method in the factoextra package. (C) Survival analysis of GSE28735 and GSE62452 by clusters. (D) Identification of the value of grouping by silhouette width plots. (E) Visualization of each cluster using the NMF method.
