# Supplementary figures and images for: Identification and Validation of Three PDAC Subtypes and Individualized GSVA Immune Pathway-Related Prognostic Risk Score Formula in Pancreatic Ductal Adenocarcinoma Patients
Source: J Oncol. 2021 Dec 27;2021:4986227. doi: 10.1155/2021/4986227 (PMC8723862; doi:10.1155/2021/4986227)

Optimal number of clusters

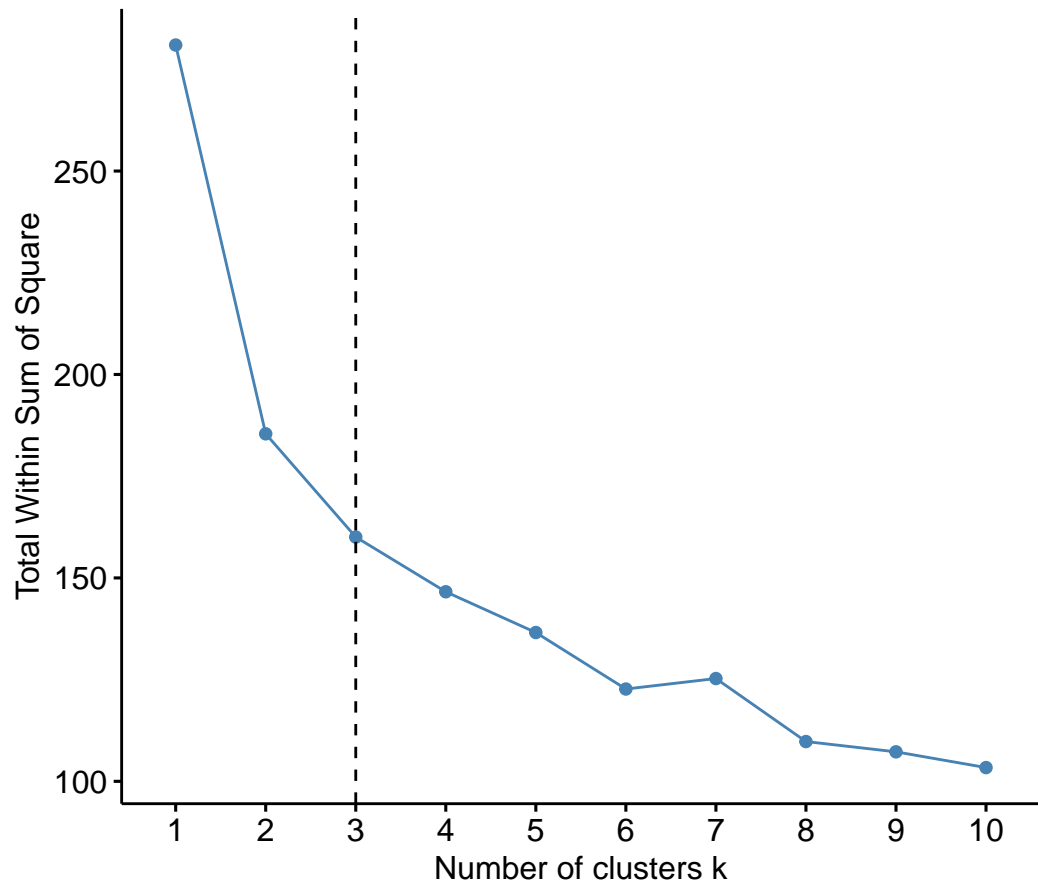

Supplement: Supplementary Materials — Supplementary Figure 1: (A) identification of best cutoff of cluster. (B) The distribution of each cluster by the PCA method in the factoextra package. (C) Survival analysis of GSE28735 and GSE62452 by clusters. (D) Identification of the value of grouping by silhouette width plots. (E) Visualization of each cluster using the NMF method. [file 4986227.f1.zip › 4986227.f1/Suplementary material Figure 1A.pdf]

## Cluster plot

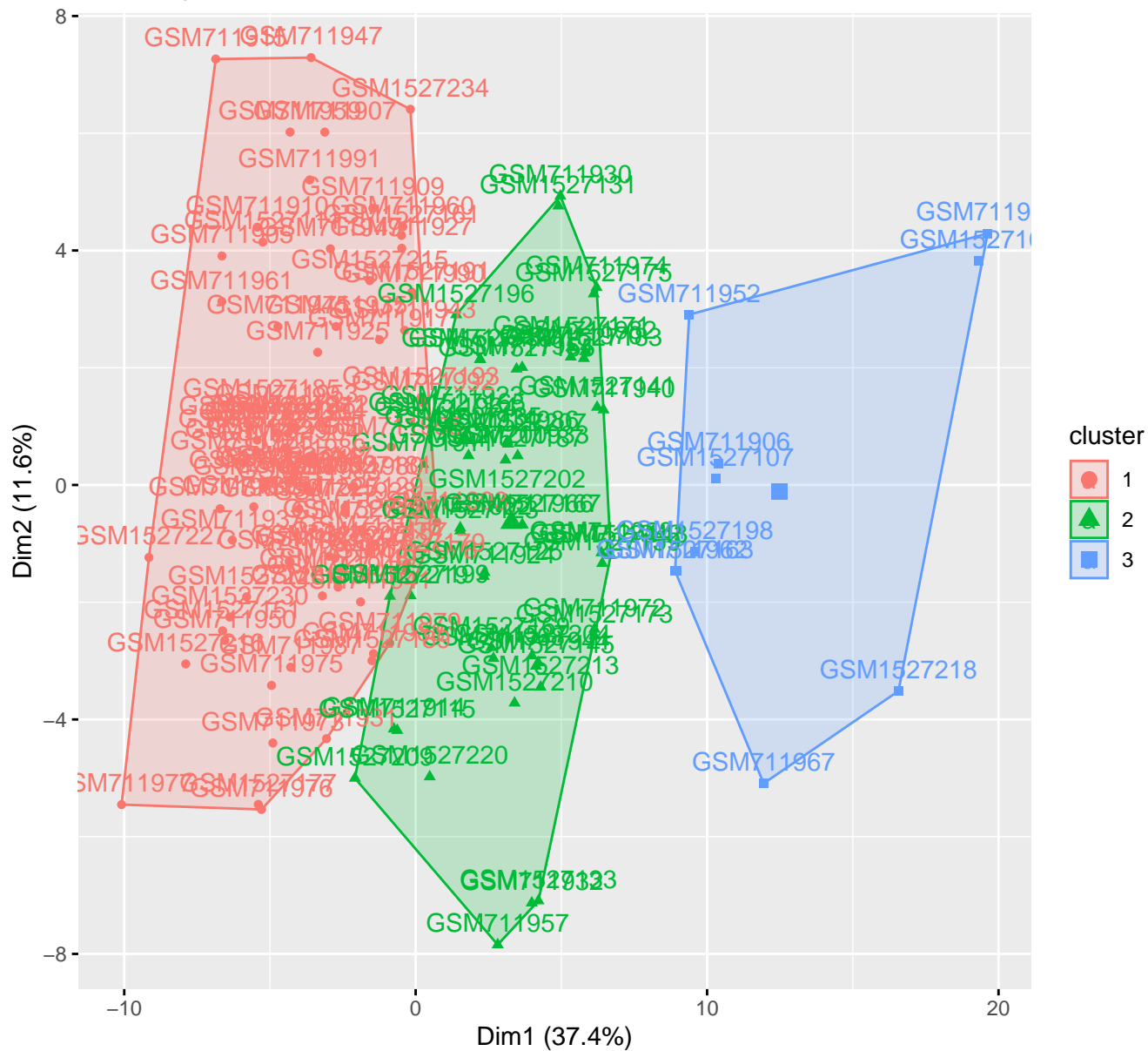

Supplement: Supplementary Materials — Supplementary Figure 1: (A) identification of best cutoff of cluster. (B) The distribution of each cluster by the PCA method in the factoextra package. (C) Survival analysis of GSE28735 and GSE62452 by clusters. (D) Identification of the value of grouping by silhouette width plots. (E) Visualization of each cluster using the NMF method. [file 4986227.f1.zip › 4986227.f1/Suplementary material Figure 1B.pdf]
